# Recent Advances in the Synthesis of N-Containing Heteroaromatics *via* Heterogeneously Transition Metal Catalysed Cross-Coupling Reactions

**DOI:** 10.3390/molecules16065241

**Published:** 2011-06-23

**Authors:** Laurent Djakovitch, Nelly Batail, Marie Genelot

**Affiliations:** Institut de Recherches sur la Catalyse et l’Environnement de Lyon (IRCELYON), Université de Lyon, CNRS, UMR 5256, 2 avenue Albert Einstein, Villeurbanne F-69626, France

**Keywords:** indoles, quinolones, dihydro*iso*quinoline-3-ones, indoxyls, heterogeneous transition metal catalysts, palladium, one-pot reactions, Larock reaction, C–H arylation, alkenylation

## Abstract

N-containing heteroaromatics are important substructures found in numerous natural or synthetic alkaloids. The diversity of the structures encountered, as well as their biological and pharmaceutical relevance, have motivated research aimed at the development of new economical, efficient and selective synthetic strategies to access these compounds. Over more than 100 years of research, this hot topic has resulted in numerous so-called “classical synthetic methods” that have really contributed to this important area. However, when the selective synthesis of highly functional heteroaromatics like indoles, quinolones, indoxyls, *etc.* is considered these methods remain limited. Recently transition metal-catalysed (TM-catalysed) procedures for the synthesis of such compounds and further transformations, have been developed providing increased tolerance toward functional groups and leading generally to higher reaction yields. Many of these methods have proven to be the most powerful and are currently applied in target- or diversity-oriented syntheses. This review article aims at reporting the recent developments devoted to this important area, focusing on the use of heterogeneous catalysed procedures that include either the formation of the heterocyclic ring towards the nuclei or their transformations to highly substituted compounds.

## 1. Introduction

Heteroaromatics are important (sub)structures present in numerous natural or synthetic alkaloids that finding application in medicinals, agrochemicals or cosmetics [1,2,3,4,5]. Among the molecules belonging to this class of compounds, condensed heteroaromatics containing at least one nitrogen atom like indoles and quinolones are undoubtedly the most relevant as they usually affect human health.

The rich structural diversity encountered in these compounds, in addition to their biological and pharmaceutical relevance, have motivated more than 100 years of research aiming at developing economical, efficient and selective synthetic strategies for these compounds [6,7,8,9,10,11,12,13]. For example, as far as indoles are concerned, these methodologies include the Fisher synthesis from aryl hydrazones [9,14], the Batcho-Limgruber synthesis from *o*-nitrotoluenes and dimethylformamide acetals [15], the Gassman synthesis from *N-*haloanilines [16,17] or the Madelung cyclisation of *N*-acyl-*o*-toluidines [18]. For quinolones, a less developed area, the most remarkable methodologies include the condensation of anilines with β-ketoesters followed by cyclisation [10,11,12] or the heterocyclisation of 2-aminochalcones [13]. The classical approaches generally reported for indoxyl synthesis include the condensation of *o*-nitrobenzaldehydes with cyclic ketones under basic conditions [19], the base induced intramolecular cyclisation of *o*-azidophenyl *sec*-alkylketones [20] or the acid promoted cyclisation of 2-diazo-2'-(*p*-tolylsulphonylamino)acetophenones [21].

These stoichiometric strategies, while successful and commonly applied in the fine chemical industry, suffer from a low structural diversity and are thus not suitable for the selective synthesis of highly functionalized compounds. As a solution, several transition metal catalyzed procedures dedicated either to the construction or the transformation of such heterocycles have been reported. For instance, the synthesis of indoles has been achieved by palladium-induced cycloadditions of 2-haloanilines with terminal or internal alkynes [22], intra- or intermolecular reactions of 2-alkynyl anilides with aryl- or alkylhalides [23]. SImilarly the preparation of quinolones by palladium catalyzed cascade carbonylation-allene insertion [24,25], or palladium catalyzed carbonylative coupling of 2-iodoanilines with arylacetylenes [26,27,28,29] have been described. The transformation of such heterocycles relies mainly on the selective functionalisation of the indole ring including arylation [8] or alkenylation [30]. These methods provide generally an increased tolerance toward functional groups and higher chemical yields [24,25,26,27,28,29,31,32,33,34,35,36,37,38,39,40,41,42] and are currently applied in target- or diversity-oriented syntheses at the laboratory scale [22,26,27,43,44,45,46]. Comparatively, concerning indoxyls, only few transition metal catalyzed procedures have been reported [47,48,49,50].

However, whatever the target nucleus, these procedures suffer from several drawbacks, the main being unacceptable metal contamination of the product, generally over the accepted limits as expressed in the medicinal regulations that often prevents further industrial development of these methods [51]. To solve such a situation, several research groups have developed alternative methodologies based on the use of heterogeneous catalysts, advantageous with respect to environment and economy, offering viable procedures at both the laboratory and the industrial scale. These methodologies are presented in this review article whose purpose is not to be exhaustive but rather to report the very recent progress aiming at developing improved processes for the synthesis of N-containing heteroaromatics by replacing homogeneous Pd- or Cu-catalysts commonly used by either commercially available or specifically designed heterogeneous Pd-catalysts.

## 2. Selective Transformation of Pre-Existing N-Containing Heterocycles

**Scheme 1:**
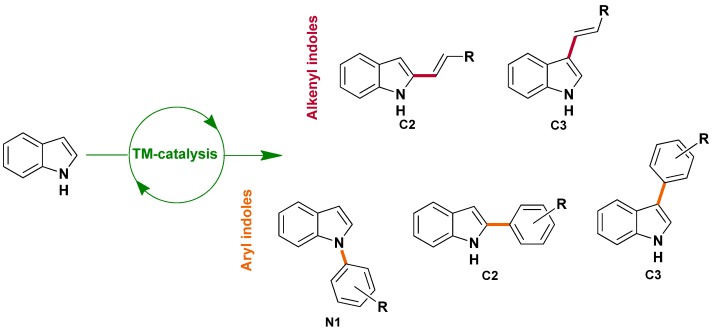
Selective transition metal (TM)-catalyzed transformation of pre-existing indoles.

This area is mainly devoted to the transformation of indole nuclei, probably due to their significance in medicinal chemistry. While all positions of the nucleus could be modified, most of these transformations involved the selective C_2_/C_3_ functionalisation of the pyrrole ring and concerned generally either the selective arylation or the selective alkenylation (Scheme 1).

### 2.1. Selective Alkenylation of Indoles

The selective palladium catalyzed alkenylation of indole was reported by Gaunt and co-workers [30]. The methodology that uses a large excess of copper salts [mainly Cu(OAc)_2_ as palladium re-oxidant] is based on the direct palladium(II) catalyzed C–H functionalisation of free NH-indoles [52].

In order to propose an environmental and economical procedure allowing at the same time decreased metal contamination of the products our group investigated the transposition of this methodology to a fully [Pd] and [Cu] catalyzed methodology using heterogeneous catalysts.

As in these systems the redox couple Pd^(II)^/Pd^(0)^ is believed to act as the active species while the Cu^(I)^/Cu^(II)^ redox couple acts as a co-catalyst to ensure the palladium re-oxidation under an oxygen atmosphere, a situation that strongly mimics that of so-called Wacker type coupling, we explored the potential of various [Pd/Cu] supported catalysts [37,40]. During these studies we found that the easy to handle [PdCl(hp)_3_Cu]_2_ complex [53] allowed us to perform the selective C_3_-akenylation of indole under air giving comparable results to that of a [Pd(OAc)_2_/Cu(OAc)_2_] catalytic system when working in a solvent mixture DMF:DMSO (10:1) at a mild 70 °C reaction temperature (Table 1).

**Table 1:** Influence of the catalytic system on the C_3_-alkenylation of indole.^a^ 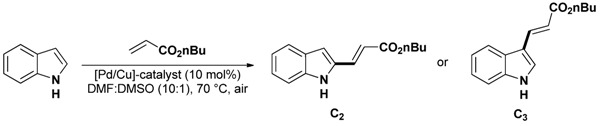

Entry	Catalyst	Conv. (20 h) ^b^	Sel. C2/C3 ^c^	Ai ^d^
1	[Pd(OAc)_2_/Cu(OAc)_2_]	52%	0/100	0.9 × 10^−3^
2	[PdCl(hp)_3_Cu]_2_	65%	0/100	0.4 × 10^−3^
3	[Pd/Cu]/NaY	60%	0/100	0.4 × 10^−3^

^a^ Reaction conditions: 3 mmol indole, 10 mol % Pd-catalyst + 10 mol % Cu-catalyst or 10 mol % as heterobimetallic catalyst, 8 mL DMF/DMSO (10/1), 70 °C, air bubbling (20 mL/min); ^b^ Conversions based on unreacted indole were determined by GC (∆rel = ± 5%); ^c^ Selectivities were determined by GC on the basis of area percentage; ^d^ Initial activity (Ai) in mol.g_Pd_^−1^.min^−1^.

In a next step, this complex was immobilized in NaY zeolite by analogy to the well-described “ship-in-a-bottle” synthesis [54]. However, ICP-AES and EPR analyses performed on the resulting material demonstrated that the atomic ratio Pd/Cu = 1.4 did not respect the stoichiometry of the complex and that the coordination environment of copper(II) and the structure of the bimetallic system changed significantly during the immobilization procedure. However, this material was found to be as active as the soluble catalyst (Table 1, entries 2 and 3) and was applied successfully to a range of indoles (Table 2).

**Table 2:** C_3_-alkenylation of 2-substituted indoles.^ a^ 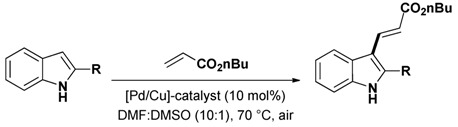

Entry	R	Conv. (1 h) ^b^	C3-selectivity ^c^
1	H	51 %	100%
2	Me	88 %	100%
3	Ph	56 %	100%
4	CO_2_Me	28 %	76% ^d^

^a^ Reaction conditions: 3 mmol indole, 10 mol % Pd(OAc)_2_, 10 mol % Cu(OAc)_2_ or 10 mol % as heterobimetallic catalyst **4**, 8 mL DMF/DMSO (10/1), 70 °C, air bubbling (20 mL/min); ^b^ Conversions based on unreacted indole were determined by GC (∆rel = ± 5%); ^c^ Selectivities were determined by GC on the basis of area percentage; ^d^ In that case the N1-alkenylated compound was formed, in 24% selectivity.

### 2.2. Selective Arylation of Indoles

Compared to the selective alkenylation of indoles, the selective N_1_, C_2_ or C_3_-arylation has known numerous developments [8], most of them being related to the use of soluble catalysts. However, in this area some research groups have devoted their work to the discovery of efficient heterogeneously catalyzed procedures.

#### 2.2.1. N_1_-arylation

The first example was reported by Reddy and co-workers using cellulose supported Cu^(0)^-catalyst. However, the procedure was limited to the phenylation of indole in a fair 60% yield using iodobenzene [indole (2 mmol), iodobenzene (1.0 equiv.), K_2_CO_3_ (2 equiv.), Cu-catalyst (*ca*. 2 mol %), DMSO, 130 °C, 24 h] [55].

Choudary and co-workers reported the use of [Cu]/NaY zeolite for the N-arylation of various imidazoles and pyrroles, including the N**_1_**-arylation of indoles. Under conditions similar to those used by Reddy [*i.e.*: indole (1.2 mmol), aryl halide (0.8 equiv.), K_2_CO_3_ (2 equiv.), Cu/NaY-catalyst (*ca*. 10 mol %), DMF, 120 °C, 24 h], quantitative yields were achieved when using either 4-iodotoluene or 4-bromotoluene [56].

**Scheme 2:**
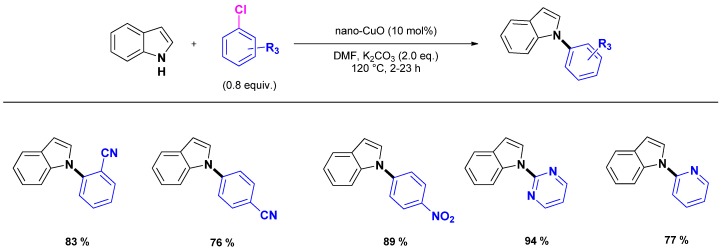
Heterogeneously copper oxide catalyzed N_1_-arylation of indoles.

The use of nanocrystalline CuO as a recoverable heterogeneous catalyst was reported by Kantam and co-workers [57]. Remarkably, the catalyst is highly effective for the coupling of activated aryl chlorides and was applied to the N_1_-arylation of various heterocycles including the indole nucleus (Scheme 2). The catalyst could be recovered by simple centrifugation and reused however with deactivation. The origin of this deactivation was not found in a structure modification of the catalyst as TEM experiments performed both on the fresh and the reused catalyst indicated that the size and the shape of the nanoparticles did not change upon reuse. 

Similarly, Punniyamurthy and co-workers reported the use of commercially available CuO nanoparticles as catalyst for the N_1_-arylation of various amines, including indole [58]. The authors compared the activity of homogenous and heterogeneous conditions. They found that CuO nanoparticles was the best catalyst compared to CuSO_4_, Cu(OAc)_2_ or CuO powder. Applied to the N_1_-arylation of indole with iodobenzene, the optimized reaction conditions [indole (1.2 mmol), iodobenzene (0.8 equiv.), KOH (0.8 equiv.), nano-CuO (1.3 mol %), DMSO, 110 °C, 3.5 h] gave the expected compound in 94% yield.

Alternatively, Chen and co-workers reported a procedure using Cu_2_O as catalyst under very similar reaction conditions [indole (1.5 equiv.), iodobenzene (1.0 mmol), KOH (1 equiv.), Cu_2_O (10 mol %), DMSO, 120 °C, 24 h] to provide *N*-phenylindole in 90% yield [59].

**Scheme 3:**
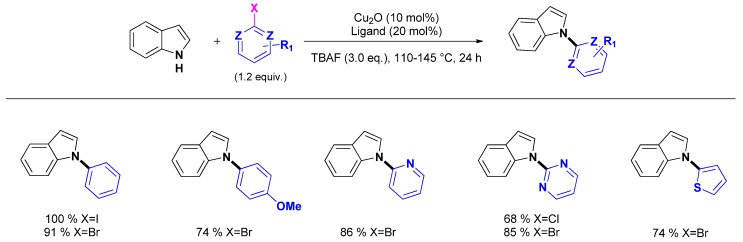
“Heterogeneously-like” N_1_-arylation of indole by Cu_2_O nanoparticles associated to 1,10-phenanthroline.

The use of Cu_2_O was also reported by Li and co-workers who evaluated the activity of various morphologies of Cu_2_O nanoparticles (cubic, octahedral, bulky…) associated to phenanthroline ligands. Cubic Cu_2_O nanoparticles/1,10-phenanthroline was found to give the best results and was applied to the N_1_-arylation of indole with various aryl halides (Scheme 3) [60]. While the catalytic system seems to answer to heterogeneous catalysts, no data concern the true homo- or heterogeneous nature of the catalytic species.

Recently, Lamaty and co-workers described the use of PEG_3400_-Cu_2_O/Cs_2_CO_3_ as an efficient and recyclable catalytic system for the selective *N*-arylation of N-heterocycles, under microwave irradiation [61]. Applied to indole, the procedure gave good to high isolated yields (*i.e.*, 57%–98%) when working with aryl iodides at 150 °C after 1–2 h. TEM experiments demonstrated that the PEG polymer acts as a protective material preventing agglomeration of Cu_2_O nanoparticles thus allowing the recycle of the PEG_3400_-Cu_2_O catalytic material for a second cycle. The authors reported that recycling procedure was limited due to low recoverability of the copper loaded polymer due to precipitation.

Wakharkar and co-workers described the use of a Cu/Fe exchange hydrotalcite. The material was prepared from a layered double hydrotalcite (LDH) by exchange with an aqueous solution of copper nitrate and ferric nitrate in order to obtain a material containing Cu^(II)^ and Fe^(II)^ species in a 3:1 ratio. This material was used for the coupling reaction of indole with either bromobenzene or 4-bromoanisole giving, respectively, 85% and 87% yield. The reactions were carried out in toluene using 10%_wt_ Cu/Fe-LDH (no data are given regarding the metal loadings in the material preventing to give the molar ratio used in the experiments), at 130 °C for 12 h [62].

An interesting approach was described by Rao and co-workers who described the use of Fe/Graphite catalysts in the selective *N*-arylation of pyrroles using aryl iodides. Working in DMSO in presence of potassium hydroxide as base, good to high isolated yields (*i.e.*, 52%–95%) were obtained, a methodology that was then extended to various N-heterocycles including indole [63]. The catalyst was efficiently reused, up to 5 runs; however, a slight deactivation was observed after the second run.

**Scheme 4:**
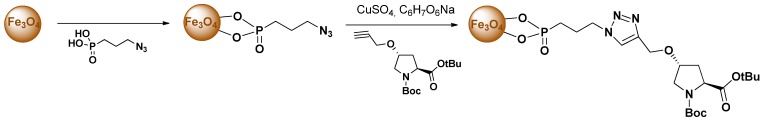
Preparation of the magnetic nanoparticle-supported L-proline.

Finally, two procedures that differ from those described above were reported recently. Alper and co-workers reported the preparation of magnetic nanoparticles-supported L-proline as recyclable and recoverable ligand in the CuI catalyzed *N*-arylation of heterocycles. The L-proline ligand bearing a terminal alkyne function was immobilized by the so-called “click chemistry” at the surface of magnetite nanoparticles (Fe_3_O_4_) modified by grafted azido groups (Scheme 4). This hybrid material was used in the N_1_-arylation of indole with 4-bromoacetophenone giving 85% yield when working in DMF at 110 °C and using 10 mol % CuI associated to a “ligand” loading of 20 mol % and Cs_2_CO_3_ as base [64]. The separated material could be recycled without adding copper iodide.

Likewise, You and co-workers reported a procedure using immobilized ionic liquid on a polystyrene matrix as a metal scavenger for catalytic applications [65]. The material was prepared by radical copolymerisation of styrene, 1,4-divinylbenzene and 1,2-dimethyl-3-(4-vinylbenzene)imidazolium chloride. For catalytic applications, the remaining chloride ions were exchanged through L-proline moiety. The resulting material showed excellent capacity in trapping transition metals, and particularly copper. It was used in the CuI catalyzed *N*-arylation of heterocycles and was applied in the N_1_-arylation of indole with iodo- and bromobenzene under classical reaction conditions [indole (0.5 mmol), bromobenzene (1.2 equiv.), K_2_CO_3_ (2.4 equiv.), CuI (10 mol %), “ligand” (20 mol %), DMSO, 120 °C, 60 h] giving respectively 99% and 68% yield. This procedure led to higher chemical yields than those observed in ionic liquids (IL) or under homogeneous conditions; however, this seems to result from longer reaction times (*i.e.*, 60 h *versus* 12–24 h). As in Alper’s method, the material loaded with the trapped copper species resulting from a first run could be reused; however, with decreased activity.

To the best of our knowledge, few reports mention selective N-arylation using heterogeneous Pd-catalysts. During the course of other studies related to the synthesis of indole rings, Djakovitch and Dufaud described the clean N-arylation of 2-phenylindole using the well-defined [PdCl_2_{PPh_2_-(CH_2_)_2_-SiCH_3_(O)_2_]@SBA-15 catalyst with aryl iodides [36] while aryl bromide led to C_3_-arylation.

#### 2.2.2. C_2_ and C_3_-arylation

**Scheme 5:**
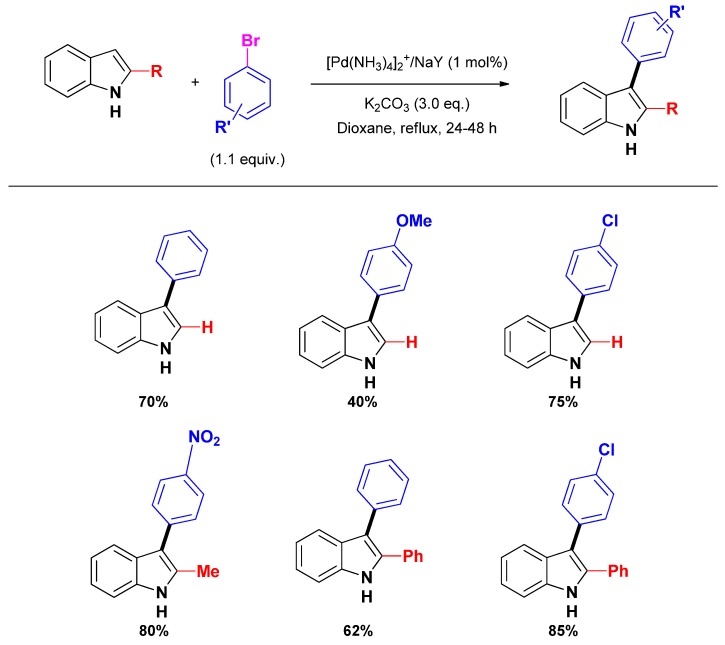
Heterogeneously palladium catalyzed selective C_3_-arylation of free (NH)-indoles with bromoarenes.

Very few articles deal with the selective C_2_ or C_3_-arylation of indoles using heterogeneous catalysts. To the best of our knowledge, the first report concerning the C_3_-arylation of indole was that by Djakovitch and co-workers, who described in 2008 the use of the well-known [Pd(NH_3_)_4_]/NaY catalyst. Remarkably, only 1 mol % of this catalyst was sufficient to achieve high conversions and moderate to good isolated yields with either indole, 2-methyl or 2-phenylindole (Scheme 5) [39]. Nevertheless, the reactivity was strongly dependent on the electronic nature of the bromoarene, electron-donating substituents giving generally the highest conversions, without rational explanation at this time.

**Scheme 6:**
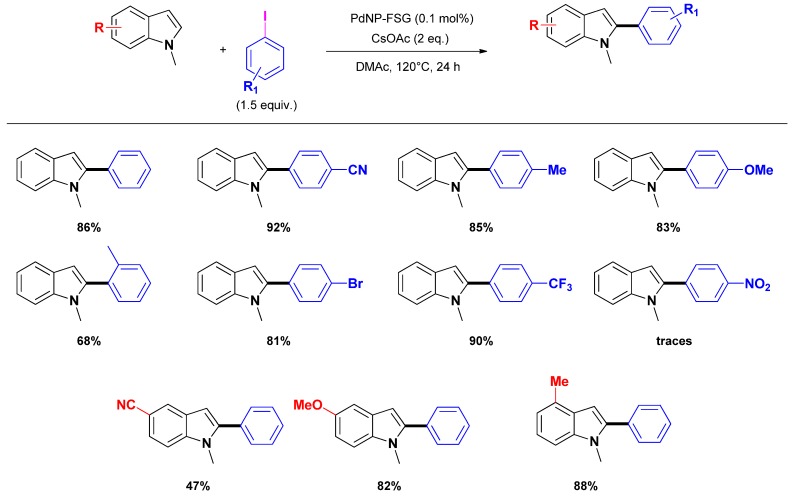
Heterogeneously palladium catalyzed selective C_2_-arylation of *N*-methyl protected indoles with Pd_NP_-FSG.

Recently, Cai and co-workers reported the use of fluorous silica-gel supported perfluoro-tagged palladium nanoparticles as an efficient and reusable catalyst for the direct C_2_-arylation of indoles [66]. The palladium nanoparticles were obtained from a sodium tetrachloropalladate solution by reduction with sodium acetate in methanol in presence of the stabilizing agent featuring long perfluorated chains as well as thio ligands. In a next step the stabilized palladium nanoparticles were immobilized on a fluorous silica-gel giving a catalytic material (denoted as Pd_NP_-FSG) that was engaged in selective C_2_-arylation of indoles giving useful to high yields (*i.e.*, 47%–85%) (Scheme 6). However, the procedure is limited to *N*-methyl protected indoles.

## 3. Selective Construction of N-Containing Heterocycles

While the selective construction of N-containing heterocycles has been strongly developed and reviewed using homogenous catalysts, that regarding implementation of transition metal based heterogeneous catalysts is quite recent and therefore less documented. The TM-catalyzed selective construction of N-containing heterocycles was mainly developed for indole synthesis; however, recent developments concerned the selective synthesis of carbonylated compounds like quinolones and indoxyls.

### 3.1. Selective Syntheses of Indoles

The methodologies developed on the basis of heterogeneous catalytic materials can be divided in two groups: 1) the one-step procedures, mainly relying on the well documented Larock indole synthesis; 2) the one-pot procedures requiring, at least, two steps involving generally a traditional cross-coupling followed by heteroannulation, that can be eventually followed by further transformations.

#### 3.1.1. Through one-step procedures

**Scheme 7:**
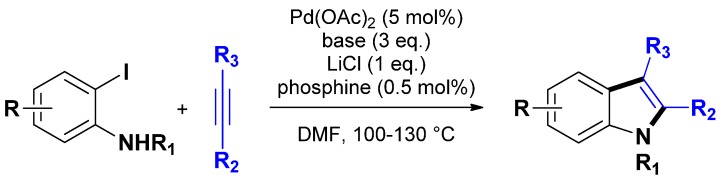
*Original* Larock indole synthesis [44].

The denomination “one-step procedure” for indole synthesis refers generally to the Larock synthesis of 2,3-functionnalized indoles, at least, when transition metal catalysts are involved [22]. This original Pd-catalyzed coupling between 2-iodoanilines and disubstituted alkynes (Scheme 7) became very popular during the last two decades [44,45]. However, the efficiency of this methodology is strongly linked to the use of soluble Pd-catalysts associated to phosphine ligands and additives as well as the use of a large excess of base and alkyne, resulting in severe practical drawbacks.

**Scheme 8:**
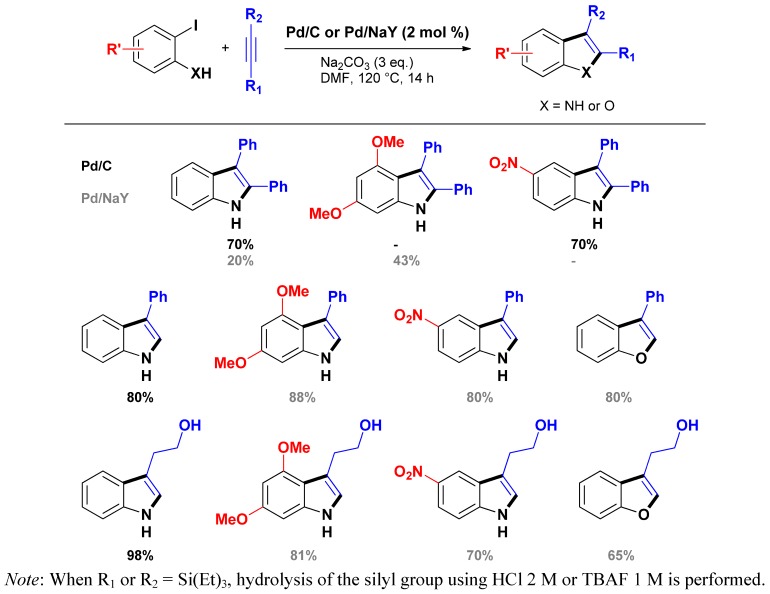
Larock heteroannulation through heterogeneous additive-free catalysis.

This situation encouraged several research groups to transpose the original Larock methodology toward the use of easily separable and reusable heterogeneous palladium catalysts. The first such methodology was reported by Batail *et al.* in 2009 [67]. With the aim of developing an additive-free (*i.e.*, ligand and salt free) Larock indole synthesis using easily separable and potentially recyclable heterogeneous palladium catalysts, the authors used in the reaction commercially available Pd/C (as Degussa type E101 NE/W from Aldrich, 10% wt on dry basis, 52% water), or the easily homemade [Pd(NH_3_)_4_]/NaY catalyst (prepared by ion exchange of a NaY zeolite using a 0.1 M aqueous solution of [Pd(NH_3_)_4_]Cl_2_ according to [68,69], 3.8% wt). Initial studies revealed that the coupling of 2-iodo-aniline with diphenylacetylene (3 equiv.) in the presence of Pd/C (2 mol %) using Na_2_CO_3_ as the base (3 equiv.) in DMF at 120 °C afforded the desired 2,3-diphenylindole in 70% isolated yield after 14 h. This result was then extended to various alkynes and 2-iodoanilines giving useful to excellent isolated yields towards the expected indoles (Scheme 8). Noticeably, the authors observed that, generally, the homemade [Pd(NH_3_)_4_]/NaY catalyst led to higher chemical yields, except when using 2-iodoaniline for which the commercial Pd/C exhibited a higher activity. Interestingly, the procedure allowed as well the synthesis of benzofurans starting from 2-iodophenol. In the same study, the authors demonstrated that the Pd/C or the [Pd(NH_3_)_4_]/NaY catalyst could be reused up to 3 and 5 times, respectively.

**Scheme 9:**
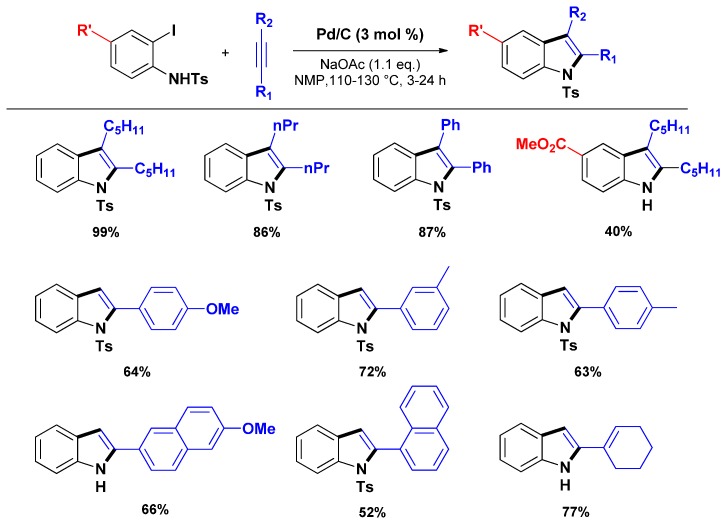
Larock heteroannulation through heterogeneous additive-free catalysis from *N*-tosyl protected anilines.

In the meantime, Sajiki and co-workers developed the use of Pd/C catalysts for the Larock heteroannulation of N-tosyl protected 2-iodoanilines with alkynes toward 2- and 2,3-substituted indoles [70]. Initially developed in presence of LiCl, the authors investigated LiCl-free procedure, providing thus a methodology close to the one reported by Batail *et al.* [67] (Scheme 9).

**Scheme 10:**
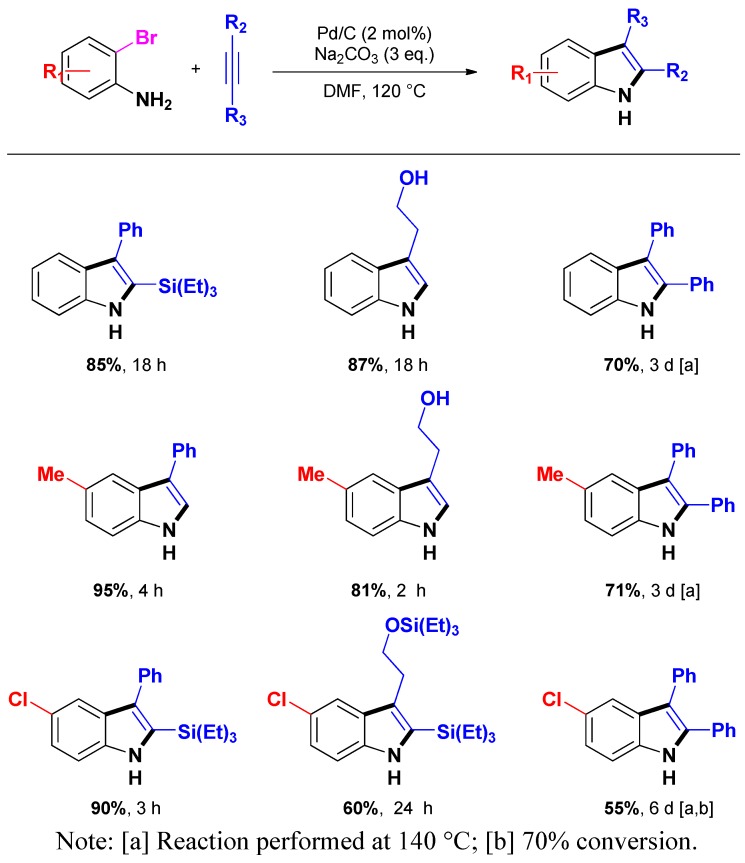
Larock heteroannulation of 2-bromoanilines through heterogeneous additive-free catalysis.

Recently, Batail *et al.* extended their methodology to a variety of substituted 2-bromoanilines and alkynes [71]. Remarkably, despite the lower reactivity of bromoaniline derivatives [72,73], indoles were obtained in good to high yields (55%–95%) enhancing the efficiency and the versatility of this heterogeneous procedure (Scheme 10). Notably, in spite of a higher temperature (*i.e.*, 140 °C) and longer reaction times inherent to the use of diphenylacetylene, good yields of the targeted indoles were obtained as common side reactions for this substrate (*i.e.*, multiple insertions or amination/dimerization of the substrates) were almost suppressed.

**Figure 1:**
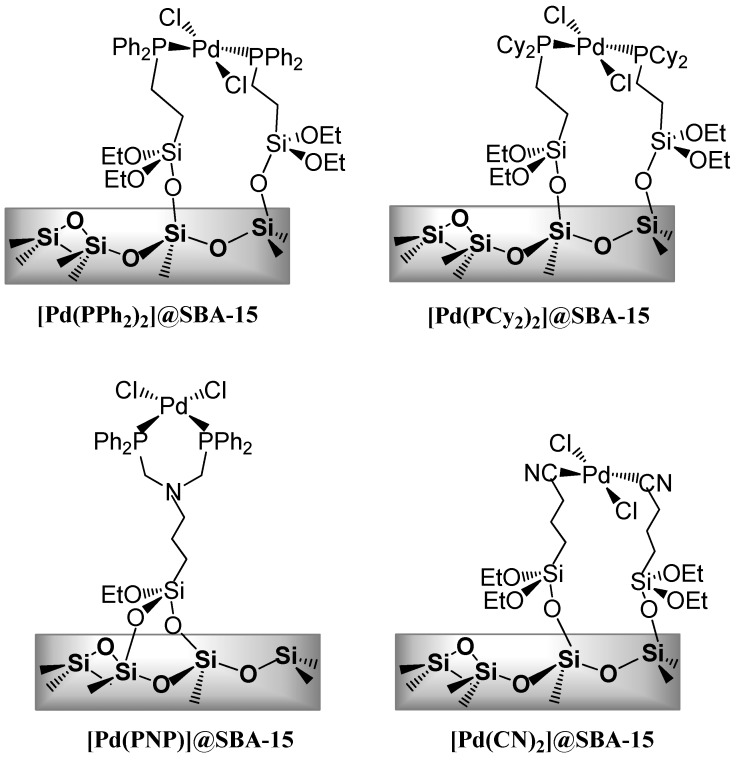
Targeted palladium catalysts based on SBA-15 silica materials generally denoted as [Pd]@SBA-15.

Alternatively, to face the lack of reactivity of some substrates, Djakovitch and Dufaud reported the preparation of palladium-containing mesostructured hybrid materials bearing either cyano ligands, monodentate or chelating phosphine linkers with various steric and electronic properties (Figure 1) [74]. As expected, these heterogeneous catalysts demonstrated higher activity than the commercially available Pd/C or the homemade [Pd(NH_3_)_4_]/NaY catalyst (*i.e.*, 1.6–3.8 mmol.mmol_Pd_^−1^.min^−1^ *versus* 0.5–1.3 mmol.mmol_Pd_^−1^.min^−1^) delivering, for example, 3-phenyl-2-(triethylsilyl)-1*H*-indole in 80%–85% isolated yields. Several experiments demonstrated that these hybrid materials tend to leached palladium species (probably active in the evaluated reaction) in the solution without affecting the recyclability of these catalysts that can be reused up to five runs giving generally quantitative conversions. However, this apparent high reusability is related to increased reaction times from 2 h to 14 h in order to circumvent a noticeable deactivation after the first run (*i.e.*, after 2 h; 30% conversion for the 2nd run *versus* 85% for the 1st run).

#### 3.1.2. Through one-pot multi-step procedures

**Scheme 11:**
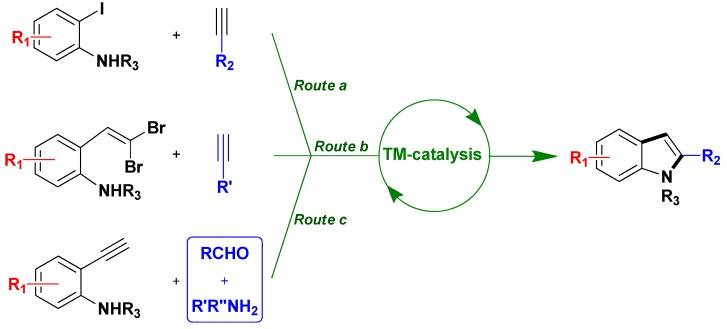
Heterogeneously TM-catalyzed routes toward indoles.

The multi-step synthesis of indole became very popular since the developments of palladium catalyzed methodologies [35]. Generally, homogeneous catalysts are applied (either as palladium salts associated to *free* ligands, or as palladium complexes); however, during the last ten years some authors initiated heterogeneously palladium catalyzed one-pot synthesis of indoles (Scheme 11).

Obviously, Djakovitch and co-workers contributed strongly to this area. Starting in 2004, these authors reported the first one-pot indole synthesis catalyzed by heterogeneous bimetallic [Pd-Cu] catalysts (*i.e.*, [PdCl(hp)_3_Cu]_2_/SiO_2_, [PdCl(hp)_3_Cu]_2_/Al_2_O_3_, [PdCl(hp)_3_Cu]_2_/NaY, [Pd(II)-Cu(II)]/SiO_2_, [Pd(II)-Cu(II)]-NaY) [32]. The procedure is based on domino reactions (Scheme 11, route a), involving a Sonogashira coupling reaction followed by intramolecular heteroannulation delivering thus quantitatively the expected 2-phenylindole under standard reaction conditions (*i.e.*: 5 mmol 2-iodoaniline, 5 mmol phenylacetylene, 15 mmol Et_3_N, 2 mol % Pd-catalyst, 15 ml DMF/H_2_O 1:1, 100 °C, 3 h). While the catalysts based on the heterobimetallic [PdCl(hp)_3_Cu]_2_ complex showed the highest activities, the easy to prepare and handle [Pd(II)-Cu(II)]/SiO_2_, [Pd(II)-Cu(II)]-NaY (obtained from the corresponding loaded metal-tetramine complex precursors by calcination under air) represent valuable alternatives. Interestingly, these heterogeneous catalysts, while deactivated during the extraction procedure in air, remain highly active and selective when used in a continuous manner. The same year, these authors extended the procedure to the use of commercially available Pd/C catalyst associated to CuI [33].

**Scheme 12:**
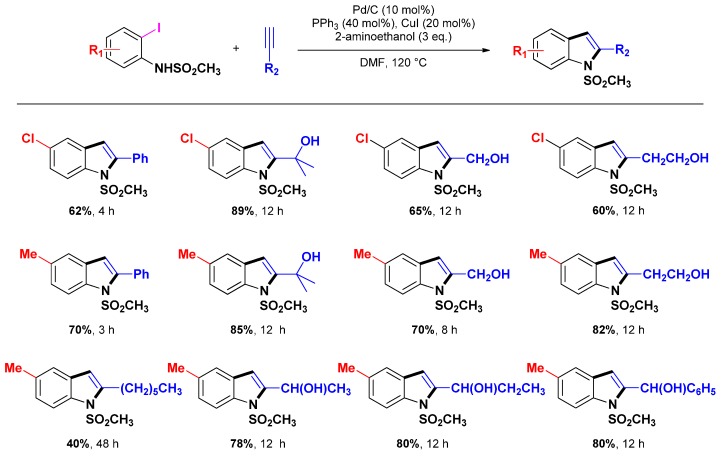
One-pot synthesis of indole following Pal *et al.* [75].

In the meantime Pal *et al.* reported a [Pd/C + CuI]-mediated synthesis of 2-substituted indoles in water [75]. The procedure is very general and proceeds *via* a tandem Pd/C mediated coupling/5-endo-dig cyclization of terminal alkynes with *N*-Methylsulfonyl *o*-iodoanilides that is required in order to observe high conversions. Additionally, the reaction must be carried out using PPh_3_ and CuI as co-catalysts and 2-aminoethanol as a base (Scheme 12). Some years later, the authors extended the scope of the procedure to other indoles [76].

A real improvement was made at the end of 2004 with the report of copper-free procedures. Developing heterogeneous palladium catalyzed Sonogashira reactions [77], Djakovitch and Rollet described the use of [Pd(NH_3_)_4_]/NaY or [Pd(NH_3_)_4_]/(NH_4_)Y catalysts for the selective synthesis of 2-phenylindole. Under mild reaction conditions (*i.e.*: 5 mmol aryl or vinyl halide, 8 mmol alkyne, 10 mmol Et_3_N, 1 mol % [Pd(NH_3_)_4_]/MY, 80 °C, DMF/H_2_O (4/1)) quantitative formation of the expected indole was observed leading to high 72% isolated yield [34].

**Scheme 13:**
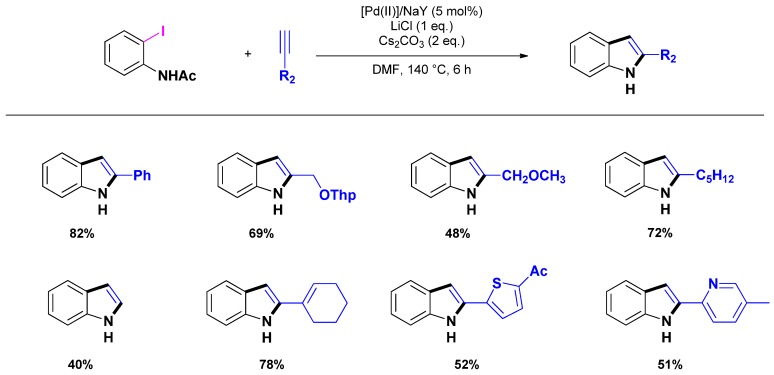
One-pot synthesis of indole catalyzed by [Pd(II)]/NaY following Yum *et al.* [78].

The same year, Yum and co-workers reported a Pd-mediated synthesis of 2-substituted indoles using [Pd(II)]/NaY (obtained by calcination of [Pd(NH_3_)_4_]/NaY) [78]. While efficient, as illustrated by moderate to high isolated yields (*i.e.*, 40%–80%), the methodology requires the use of *N*-acetyl *o*-iodoanilide and cesium carbonate as base in the presence of stoichiometric amount of LiCl to achieve competitive conversions (Scheme 13).

**Scheme 14:**
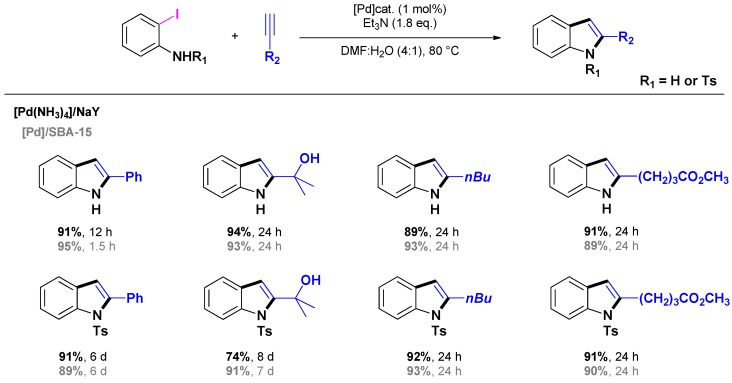
One-pot synthesis of indole catalyzed by [Pd(NH_3_)_4_]/NaY or [Pd]/SBA-15 following Djakovitch *et al.* [36].

To overcome such limitations related to the need of N-protected iodoanilines, Djakovitch and Dufaud described the preparation of [Pd(PPh_2_)_2_]@SBA-15 (Figure 1) catalyst and compared its activity to that of [Pd(NH_3_)_4_]/NaY, Pd(OAc)_2_ and “Beller-Herrmann Palladacycle” {Pd[P(*o*-C_6_H_4_CH_3_)_2_-(*o*-C_6_H_4_CH_2_)(CH_3_CO_2_)]}_2_ [36]. Generally high conversions and selectivities (>89% yield) leading to moderate to high isolated yields were obtained using 1 mol % [Pd]-catalyst under standard reaction conditions (DMF/H_2_O (4/1), 80 °C) (Scheme 14). In all cases, under author's reaction conditions, heterogeneous catalysts were found to be more active than homogeneous ones which suffer from deactivation by formation of palladium black. Additionally, the [Pd(PPh_2_)_2_]@SBA-15 catalyst exhibited a slightly higher activity than the [Pd(NH_3_)_4_]/NaY one; however, recycling experiments demonstrated that the former catalyst could be reused up to five runs while the [Pd(NH_3_)_4_]/NaY catalyst was fully deactivated after the 3^rd^ run. Such a difference was attributed to the rate of Pd-leaching which is negligible with [Pd(PPh_2_)_2_]@SBA-15 whereas it is pronounced when engaging [Pd(NH_3_)_4_]/NaY in the reaction. Interestingly, the authors demonstrated the opportunity to synthesize 2,3-functionalised indoles by a further direct arylation using the [Pd(PPh_2_)_2_]@SBA-15 catalyst. However, the procedure is not very efficient as a low 35% yield was obtained after 12 days of reaction.

**Scheme 15:**
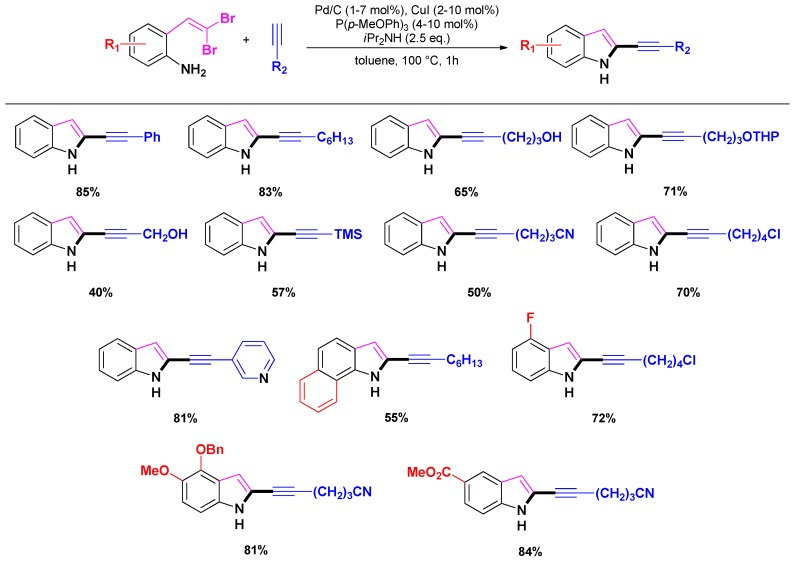
One-pot synthesis of indole catalyzed by [Pd(NH_3_)_4_]/NaY or [Pd]/SBA-15 following Lautens and co-workers [79].

Recently, Lautens and co-workers reported an interesting approach to access 2-alkynyl indoles *via* a tandem Cu- and Pd-catalyzed cross-coupling procedure (Scheme 11, route b) [79]. The methodology involves the use of readily accessible *ortho*-*gem*-dibromovinylanilines (and phenols) that led to cyclisation toward the expected indoles (and benzofurans) through a tandem Ullman C-N/Sonogashira reaction. In this sequence, it is believed that copper catalyzes the Ullman C-N coupling toward 2-bromoindoles, while the palladium catalyst activates the remaining Sonogashira reaction. Noticeably, a screening of catalysts and bases revealed a combination of Pd/C, CuI, P(*p*-MeOPh)_3_ and di*iso*propylamine as the most effective system to prepare a range of 2-alkynylindoles under relatively mild reaction conditions (Scheme 15). As often reported, the authors considered that the reaction occurs through leached (dissolved) Pd-species [34,37,80,81] as the nature of the support did not affect the reaction.

**Scheme 16:**
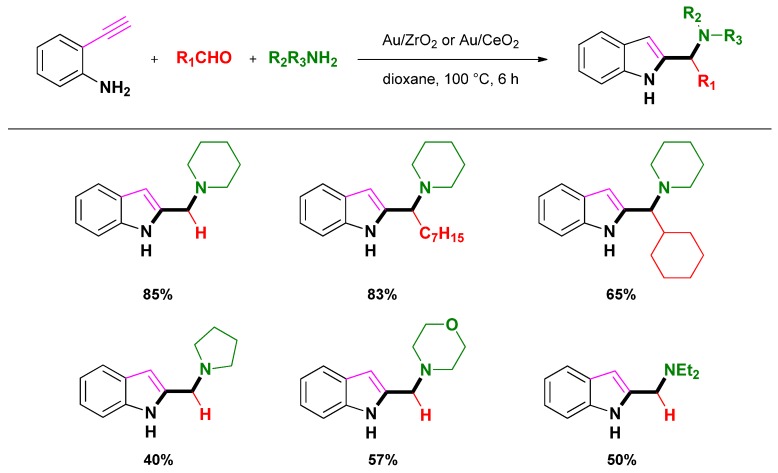
Three-component one-pot synthesis of indole catalyzed by Au/ZrO_2_ or Au/CeO_2_ following Corma et co-workers. [82].

Corma and Zhang reported the use of gold(III) nanoparticles supported on ZrO_2_ or CeO_2_ in a three-component reaction featuring the cyclization of an aldehyde, an amine, and 2-ethynylaniline (Scheme 11, route c) toward the synthesis of indoles (Scheme 16) [82]. The synthetic pathway involved the alkynylation of iminium ions through C-H activation of alkynes followed by reductive cyclization. The overall process displayed high turnover frequencies and numbers using only 0.35 mol % Au. Moreover, the air-stable catalysts (Au/ZrO_2_ or Au/CeO_2_) could be easily recovered by filtration and reused at least three times with only a slight decrease of the catalytic activity. The authors speculated that the reaction mechanism could be the same as the one involved with cationic Au(III)-species under homogeneous conditions, the Au(III)-species being stabilized by nanocrystalline CeO_2_ or ZrO_2_.

**Scheme 17:**
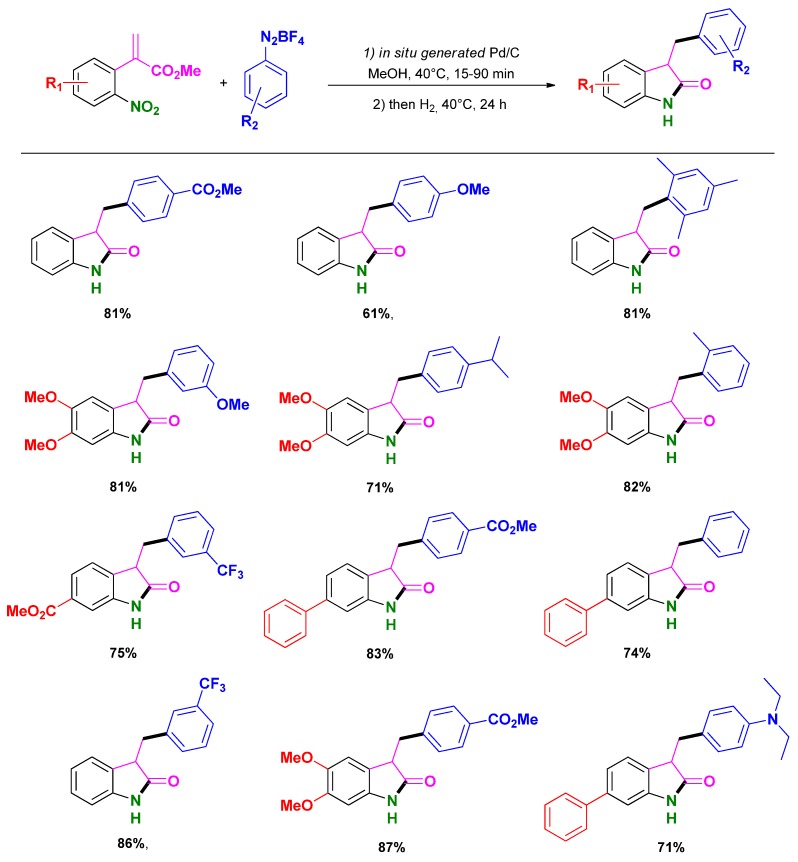
HRC one-pot synthesis of oxindole catalyzed by Pd/C following Felpin and co-workers [83].

Recently, Felpin and co-workers reported the synthesis of oxindoles from 2-(2-nitrophenyl)acrylates and aryldiazonium salts through a tandem Heck/Reduction/Cyclization (HRC) sequence catalyzed by palladium [83,84,85]. The methodology initially developed using Pd/C catalyst was improved by generating *in situ* the catalytic material from palladium acetate and charcoal. The optimized conditions (*i.e.*, acrylates (1 mmol), aryldiazonium salt (1.2 mmol), Pd(OAc)_2_ (5 mol %), charcoal (45 mg), MeOH (5 mL), 40 °C, 15–90 min; then H_2_, 40 °C, 24 h.) for this HRC sequence gave access to various oxindoles in good to high yields (Scheme 17). Interestingly, the only waste produced during the Heck coupling (*i.e.*, HBF_4_) acts as a co-catalyst in the subsequent reduction-cyclization step becoming thus beneficial to the overall process.

### 3.2. Selective Syntheses of 2-Quinolones

**Scheme 18:**
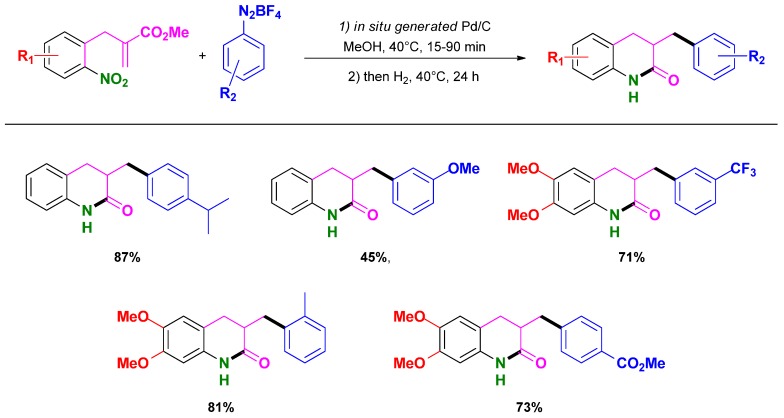
HRC one-pot synthesis of 2-quinolone from 2-(2-nitrophenyl)acrylates and aryldiazonium salts catalyzed by Pd/C following Felpin and co-workers [86].

**Scheme 19:**
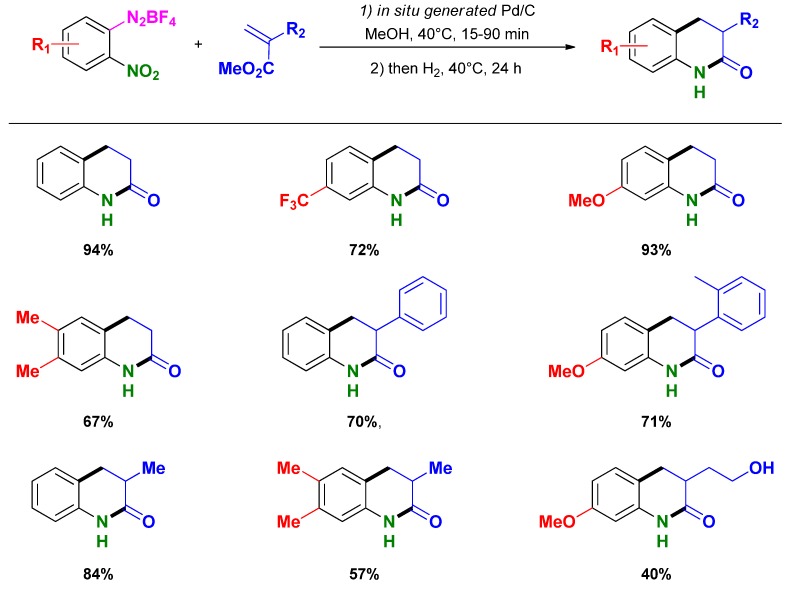
HRC one-pot synthesis of 2-quinolone from 2-nitrobenzenediazonium salts and acrylates catalyzed by Pd/C following Felpin and co-workers [86].

Following their HRC methodology, Felpin *et al.* reported the selective synthesis of 2-quinolones using either heterogeneous catalysts or mixed homogeneous/heterogeneous catalysts with charcoal as support [86]. The overall reaction sequence proceeds under mild conditions (*i.e.*, acrylate (1 mmol), aryldiazonium salt (1.2 mmol), Pd(OAc)_2_ (5 mol %), charcoal (110 mg), MeOH (5 mL), 40 °C, 30–90 min, then H_2_, 40 °C, 24 h) providing good to high isolated yields starting either from 2-(2-nitrophenyl)acrylates and aryldiazonium salts (Scheme 18) or 2-nitrobenzenediazonium salts and acrylates (Scheme 19).

### 3.3. Selective Syntheses of 4-Quinolones

Among the different methods described for the synthesis of 2-substituted-4-quinolones [87,88,89,90,91,92,93,94,95,96,97,98,99,100,101,102,103], the palladium catalyzed carbonylative coupling of 2-iodoanilines with arylacetylenes, initially reported by Torii and co-workers [26,104], appears to be the most versatile. These authors obtained good yields towards the expected compounds using homogeneous [PdCl_2_(PPh_3_)_2_] or [PdCl_2_(dppf)] (5 mol %) in presence of an excess of diethylamine which plays a key role in the cyclization step. However, as reported elsewhere [29], we observed that diethylamine tends to give huge amount of undesired by-products upon side reactions with the starting reactants.

**Scheme 20:**
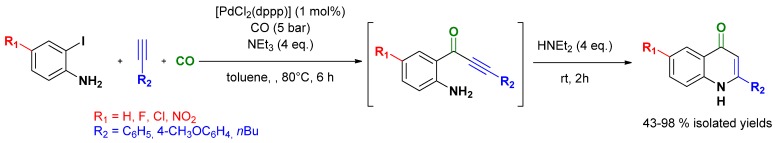
One-pot two-step multi-catalyzed synthesis of 4-quinolone following Djakovitch and co-workers [105].

In order to elucidate factors controlling the selectivity toward 2-substituted-4-quinolones, our group performed extensive studies varying the nature of the Pd-catalysts, ligands, bases, additives and solvents. These studies revealed that 2-substituted-4-quinolones could be selectively obtained through a *one-pot two-step multi-catalysis* using sequentially [PdCl_2_(dppp)] and HNEt_2_ as catalysts (Scheme 20) [105].

**Scheme 21:**
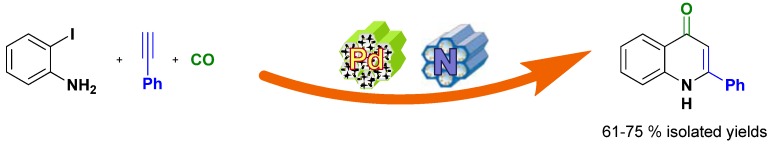
One-pot heterogeneous tandem catalysis {[Pd(PNP)]@SBA-15/[NH_2_(CH_2_)_3_]@SBA-3} to access 4-quinolones following Djakovitch and co-workers [106].

Thus, we extended the procedure to the use of heterogeneous catalysts associating the [Pd(PNP)]@SBA-15 catalyst (Figure 1) to a grafted amine catalyst as [NH_2_(CH_2_)_3_]@SBA-3 in a mechanical mixture that allowed, for example, the selective synthesis of 2-phenyl-4-quinolone in a suitable 61% isolated yield (Scheme 21) [106]. Interestingly, such an approach resulted in a strong decrease of palladium contamination in the final products as only 3 to 5 ppm of palladium was found in the crude 4-quinolones while 40 ppm was measured when using homogenous catalytic system.

### 3.4. Selective Syntheses of Indoxyls

**Scheme 22:**
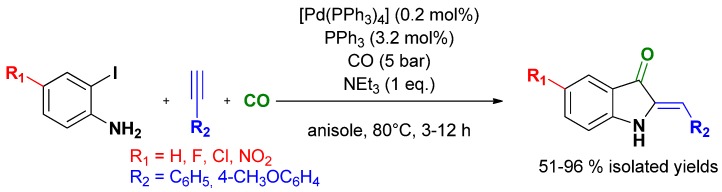
One-pot two-step multi-catalyzed synthesis of 4-quinolone following Djakovitch and co-workers [105].

During our studies toward the selective synthesis of quinolones, we observed that the cyclization step toward indoxyls was catalyzed by free phosphines. Thus [Pd(PPh_3_)_4_] used as catalyst afforded quantitatively the expected 2-benzylideneindoxyl from 2-iodoaniline, phenyl acetylene and carbon monoxide. The procedure was extended to a variety of indoxyls, mainly obtained as the (*Z*)-isomer (Scheme 22) [105].

Our attempts to transpose this procedure in a heterogeneous fashion by associating [Pd(PPh_2_)_2_]@SBA-15 to [PPh_2_(CH_2_)_2_]@SBA-15 catalysts were not successful as the by-product 2-benzyl-*1H*-indole [107] was observed in 30-71% selectivity depending on the reaction time [106]. However, the combination of the [Pd(PPh_2_)_2_]@SBA-15 catalyst with PPh_3 _allowed the selective synthesis of 2-benzylidene-indoxyl. Such a semi-heterogeneous catalytic system features a decreased metal contamination in the crude product (*i.e.*, 28 ppm compared to 94 ppm with a fully homogeneous catalytic system). While effectively reduced, the Pd-content in the crude indoxyl is still too high for a direct use in pharmaceutical applications, because of the possible stabilization of leached Pd-species in solution by soluble triphenyl phosphine.

### 3.5. Selective Syntheses of Dihydroisoquinolin-3-ones

**Scheme 23:**
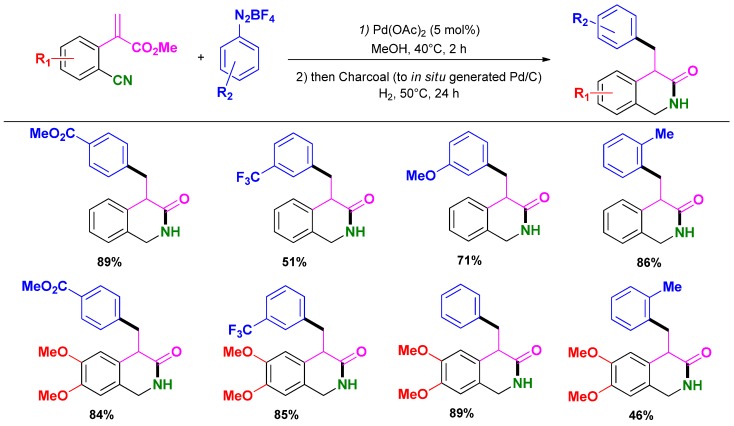
HRC one-pot synthesis of 4-benzyl-1,2-dihydroisoquinolin-3-ones following Felpin and co-workers [108].

Developing further their HRC methodology, Felpin and co-workers reported the selective synthesis of 4-benzyl-1,2-dihydroisoquinolin-3-ones from 2-(2-cyanoaryl)acrylates and aryldiazonium salt [108]. The overall reaction sequence proceeds under mild conditions (i.e. acrylate (1 mmol), aryldiazonium salt (1.2 mmol), Pd(OAc)_2_ (5 mol %), MeOH (5 mL), 40 °C, 12 h; then charcoal (110 mg), H_2_, 50 °C, 24 h) giving good to high isolated yields toward expected compounds (Scheme 23).

## 4. Conclusions

This mini-review featured recent developments in the discovery of heterogeneous TM-based catalytic procedures (mainly, palladium ones) for the synthesis or the functionalisation of N-containing heterocycles like indoles, quinolones, indoxyls or 1,2-dihydroisoquinolin-3-ones. These procedures highlight original and practical improvements resulting in reliable tools, competitive with existing methodologies and of great interest for the synthetic chemists, whether academic or industrial.

This research area, while intensively developed during the last ten years, is still in its infancy and studied by only few research groups. We believe that the operationally simple procedures described in this article represent attractive protocols, most of them contributing to sensible approaches toward greener chemistry. Obviously, the scope of this research topic is not limited to the few examples reported herein and we hope that this short review article will stimulate further works in these directions.
